# Characterization of Four Novel Bacteriophages Isolated from British Columbia for Control of Non-typhoidal *Salmonella in Vitro* and on Sprouting Alfalfa Seeds

**DOI:** 10.3389/fmicb.2017.02193

**Published:** 2017-11-15

**Authors:** Karen Fong, Brett LaBossiere, Andrea I. M. Switt, Pascal Delaquis, Lawrence Goodridge, Roger C. Levesque, Michelle D. Danyluk, Siyun Wang

**Affiliations:** ^1^Food, Nutrition, and Health, University of British Columbia, Vancouver, BC, Canada; ^2^Escuela de Medicina Veterinaria, Facultad de Ecología y Recursos Naturales, Universidad Andres Bello, Santiago, Chile; ^3^Agriculture and Agri-Food Canada, Summerland, BC, Canada; ^4^Department of Food Science and Agricultural Chemistry, McGill University, Montreal, QC, Canada; ^5^Institute for Integrative and Systems Biology, Université Laval, Québec City, QC, Canada; ^6^Department of Food Science and Human Nutrition, Citrus Research and Education Center, University of Florida, Lake Alfred, FL, United States

**Keywords:** *Salmonella*, bacteriophage, biocontrol, sprouts, food safety

## Abstract

Alfalfa sprouts have been linked to numerous North American outbreaks of *Salmonella* in recent years. Conventionally, treatments involving chlorine, heat, and irradiation are used for alfalfa seed sanitation. However, such treatments may be highly variable in their efficacy for pathogen control and/or detrimental to sprout quality, therefore negatively perceived by consumers advocating for natural alternatives. The usage of bacteriophages for pathogen control in sprouts has been previously explored, although with conflicting and inconsistent results. Lytic phages, viral predators of bacteria, represent an attractive approach as they provide several advantages compared to conventional treatments, such as their high specificity for bacterial targets and their ubiquity in nature. In this study, four *Salmonella* phages were isolated from British Columbia, Canada and characterized with respect to host range, burst size, latent period, and environmental stability to assess their potential to control *Salmonella*. Phage isolate SI1 showed the greatest host range, highest burst size and shortest latent period, greatest stability across all pH and temperatures and was the most effective in control of *S.* Enteritidis *in vitro*. Therefore, SI1 was chosen for treatment of sprouting alfalfa seeds artificially contaminated with *S.* Enteritidis with a multiplicity of infection (MOI) of ∼110 PFU/CFU. A significant (*p* < 0.05) reduction of 38.3 ± 3.0% of viable *Salmonella* cells was observed following two h of phage treatment. On days two to six of the sprouting process, reductions of *Salmonella* were also observed, but were not significant compared to the control (*p* > 0.05). It was further demonstrated that the sprout yield was not significantly (*p* > 0.05) affected by phage treatment. These results highlight the potential of phages recovered from the British Columbia environment for use as biocontrol agents against *Salmonella*, although differing efficacies *in vitro* was observed. Moreover, the effectiveness of SI1 to significantly (*p* < 0.05) control *Salmonella* on sprouting alfalfa seeds on day 1 of treatment was demonstrated. Although promising, future work should aim to optimize this treatment to achieve more effective, and longer lasting, biocontrol of *Salmonella* in sprouting alfalfa seeds.

## Introduction

Foodborne diseases caused by non-typhoidal *Salmonella* represent a significant public health burden worldwide. It is estimated that 80.3 million cases arise yearly from food products contaminated by *Salmonella*, resulting in over 100, 000 deaths (Majowicz et al., 2010). Although particular foods (e.g., poultry, eggs, and swine) have been historically classified as definitive causative agents for salmonellosis, emerging disease transmission vectors (e.g., fresh produce) have been associated with numerous outbreaks in recent years (Centers for Disease Control and Prevention [CDC], 2017).

In North America, seed sprouts are becoming increasingly popular amongst consumers as they are nutrient-dense, versatile, and relatively inexpensive (Pérez-Balibrea et al., 2008). Unfortunately, an increase in the number of *Salmonella* outbreaks has been attributed to the consumption of raw seeds sprouts (Centers for Disease Control and Prevention [CDC], 2017), with the majority of outbreaks linked to alfalfa sprouts (Proctor et al., 2001; Kumar et al., 2006). A variety of *Salmonella* serotypes have been associated with sprouts-related illnesses, including common serotypes (e.g., *S.* Enteritidis, *S.* Typhimurium and *S.* 4,[5],12:i:-) and less common serotypes (e.g., *S.* Agona, *S.* Reading, and *S.* Abony) (Centers for Disease Control and Prevention [CDC], 2017).

Contamination of seeds used for sprouts production is likely to occur in the field (Food and Drug Administration [FDA], 2015). Once contaminated, sprouting seeds provide an ideal habitat for support and growth of *Salmonella* (Fahey et al., 2006). During the sprouting process, enhanced humidity, warm temperatures, and the release of nutrients from the seed itself, result in the rapid proliferation of *Salmonella* (Fahey et al., 2006); an increase of over three log CFU/g of viable *Salmonella* during the sprouting of alfalfa seeds has been reported (Charkoswski et al., 2002).

A variety of intervention strategies are available to disinfect sprouts seeds, including the usage of chemical disinfectants (e.g., hypochlorite, calcium hydrogen peroxide), heat, and irradiation (National Advisory Committee on Microbiological Criteria for Foods [NACMCF], 1999). The reliability and consistent implementation of these treatments, however, has been questioned as sprouts-related illnesses continue to rise. In Canada, seed sanitation is not required by law (Canadian Food Inspection Agency [CFIA], 2014), although it is recommended that a seed treatment capable of attaining a minimum three-log reduction be considered (Canadian Food Inspection Agency [CFIA], 2014). However, negative consumer perceptions regarding chemical and physical treatments and its potentially negative impacts on the yield and quality of sprouts may hinder the widespread adoption of such treatments (Kim et al., 2003). Additionally, the efficacy of these treatments has been shown to be highly variable (Montville and Schaffner, 2004). For instance, the reference standard for seed disinfection, 20,000 ppm calcium hypochlorite, has resulted in variable microbial reductions of 0.51 – 6.90 log CFU/g (Ding et al., 2013). Disinfection with other chemicals alternative chemicals is also highly variable regarding their microbial kill; electrolyzed oxidizing water previously achieved reductions of 1.66 log CFU/g (Kim et al., 2003), while 5% acetic acid achieved reductions of 2.40 log CFU/g (Lang et al., 2000). Lastly, physical inactivation methods are gaining interest; soaking seeds in hot water at 85°C for 10 s was reported to achieve a 3.0 log CFU/g reduction (Bari et al., 2010). High pressure for 500 MPa for two min similarly achieved a 3.5 log CFU/g reduction (Neetoo and Chen, 2010). However, physical treatments have been shown to inhibit the germination rate and may not be commercially viable methods for disinfection (Bari et al., 2009). Therefore, alternative measures are urgently needed for effective, clean-label decontamination methods, without negatively impacting seed viability.

Bacteriophages (phages) are viral predators of bacteria (Hagens and Loessner, 2007) that have attracted considerable interest as a method for pathogen control on foods. Previous research has evaluated its use on a variety of foodstuffs, including chicken skin (Goode et al., 2003), broccoli and mustard sprout seeds (Pao et al., 2004), fresh-cut produce (Leverentz et al., 2001) and cheddar cheese (Modi et al., 2001). Phages possess several properties that render them suitable for use on food because they are: (i) highly specific, not crossing species or genus barriers; (ii) designed to kill host cells only; (iii) self-replicating and self-limiting; and (iv) ubiquitously distributed in nature (Rohwer and Edwards, 2002). Despite the range of desirable attributes, however, the usage of phages as biocontrol agents for *Salmonella* in sprouts has not been widely adopted, although there have been previous reports of similar efforts (Pao et al., 2004; Kocharunchitt et al., 2009). At an initial density of approximately seven log CFU/g of *S.* Oranienburg, relatively low log reductions of approximately one log CFU/g of *Salmonella* were achieved with phage SSP6 (Kocharunchitt et al., 2009). Additionally, Pao et al. (2004) reported a 1.50 log CFU/g reduction of *Salmonella* upon application of a bacteriophage cocktail on broccoli seeds artificially contaminated with an initial density of 7–7.5 log CFU/g. The relatively low reduced efficacies may have been due to the limited number of effective phages recovered, and/or the failure to adequately characterize phages for this particular purpose. The objectives of this study were to characterize four broad-host range *Salmonella* phages on the basis of their phenotypic and genotypic determinants, assess their infectivity against various *Salmonella* strains *in vitro*, and evaluate the efficacy of a promising phage isolate, SI1, for biocontrol of *S.* Enteritidis on alfalfa seeds throughout the sprouting process.

## Materials and Methods

### Bacterial Strains and Growth Conditions

*Salmonella enterica* serotype Enteritidis FSL S5-483 was used as the bacterial host for the phages in this study. The *Salmonella* strains used in the host range study are listed in **Table 1**. All strains were maintained at -80°C in Brain-Heart-Infusion broth (BD/Difco, East Rutherford, NJ, United States) supplemented with 20% glycerol. Working stocks were maintained on tryptic soy agar (TSA; BD/Difco, East Rutherford, NJ, United States) at 4°C for a maximum of 1 month. Prior to each experiment, fresh overnight cultures were prepared by inoculating an isolated colony into 10 ml tryptic soy broth (TSB; BD/Difco, East Rutherford, NJ, United States). Cultures were then incubated for 16 h at 37°C with gentle shaking at 170 rpm.

**Table 1:** Host ranges of SI1, SS1, SS4 and SF1.

*Salmonella* strain	SI1	SF1	SS1	SS4
*S*. Enteritidis FSL S5-483^a^ (host)	4	4	4	4
*S*. Thompson FSL S5-523^a^	0	0	0	0
*S*. Braenderup FSL S5-373^a^	0	0	0	0
*S*. Muenchen FSL S5-504^a^	0	0	0	0
*S*. Montevideo FSL S5-630^a^	0	0	0	0
*S*. Saintpaul FSL S5-649^a^	3	3	2	3
*S*. Typhimurium FSL S5-536^a^	4	2	0	3
*S*. Javiana FSL S5-406^a^	0	0	0	2
*S*. Senftenberg FSL S5-658^a^	0	0	0	0
*S*. Mbandaka FSL S5-451^a^	0	0	0	0
*S*. Agona FSL S5-517^a^	4	2	3	4
*S*. Newport FSL S5-436^a^	0	0	0	0
*S*. Typhimurium LMFS-JF-001^b^	4	4	4	4
*S*. Newport S2^c^	0	0	0	0
*S*. Newport S195^c^	0	0	0	0
*S*. Enteritidis S3^c^	4	4	4	4
*S*. Enteritidis S187^c^	3	3	4	4
*S*. Canada S30^c^	4	4	4	4
*S*. Chingola S32^c^	0	0	0	0
*S*. Luciana S43^c^	0	0	0	0
*S*. Typhimurium S189^c^	4	4	4	4
*S*. Heidelberg S191^c^	3	3	4	4
*S*. Thompson S193^c^	0	0	1	1
*S*. Thompson S194^c^	0	0	1	1
*S*. Infantis S198^c^	0	0	0	0
*S*. Javiana S200^c^	3	3	4	4
*S*. Javiana S203^c^	3	3	4	4
*S*. Saintpaul S204^c^	3	3	2	4
*S*. Saintpaul S205^c^	3	3	2	4
*S*. Muenchen S206^c^	0	0	0	0
*S*. Muenchen S207^c^	0	0	0	0
*S*. Agona S213^c^	2	2	0	0
*S*. Agona S215^c^	4	4	3	4
*S*. Oranienburg S216^c^	0	0	0	0
*S*. Hadar S219^c^	0	0	0	0
*S*. Mbandaka S236^c^	0	0	0	0
*S*. Mbandaka S238^c^	0	0	0	0
*S*. Montevideo S239^c^	0	0	0	0
*S*. Montevideo S241^c^	0	0	0	0
*S*. Bareilly S258^c^	0	0	0	0
*S*. Senftenberg S269^c^	0	0	0	0
*S*. Senftenberg S270^c^	0	0	0	0
*S*. Litchfield S272^c^	0	0	0	0
*S*. Litchfield S273^c^	0	0	0	0
*S*. Uganda S276^c^	0	0	0	0
*S*. Uganda S277^c^	0	0	0	0
*S*. Havana S286^c^	0	0	0	0
*S*. Poona S306^c^	0	0	0	0
*S*. Poona S307^c^	0	0	0	0
*S*. Ohio S316^c^	0	0	0	0
*S*. Berta S333^c^	4	1	4	4
*S*. Liverpool S346^c^	0	0	0	0
*S*. Rubislaw S348	0	0	0	0
*S*. Typhimurium S441^c^	4	4	4	4
*S*. Anatum S443^c^	0	0	0	0
*S*. Typhimurium LT2^d^	3	3	4	3
4, 5, 12:I:- FSL S5-580^a,e^	4	4	3	4
*S.* Typhimurium 14028S 1-5^e^	3	3	4	3
*S.* Typhimurium var. Copenhagen FSL S5-786^a,f^	1	1	0	0
*S*. Typhimurium SL1344^g^	3	4	4	3
*S*. Schwarzengrund FSL S5-458^a,h^	0	0	0	0

*Salmonella strains susceptible to phage infection are indicated by a +3 or +4 clearing. ^a^ILSI North America Collection; ^b^strain isolated from irrigation water in the Lower Mainland, British Columbia, Canada; ^c^the *Salmonella* Foodborne Syst-OMICS database (SalFoS) collection; ^d^Strain resistant to STR (Lilleengen, 1950); ^e^strain resistant to AMC, AMP, FOX, CHL, STR, SUF, TET; ^f^strain resistant to AMC, AMP, FOX, CRO, CHL, KAN, STR, SUF, TET; ^g^strain resistant to STR (Hoiseth and Stocker, 1981); ^h^strain resistant to AMP, CIP, NAL, SUF, TET, SXT.*

### Bacteriophage Isolation and Purification

Bacteriophages were isolated from irrigation water (*n* = 15), cattle feces (*n* = 9) and sediment obtained from the bottom of irrigation ditches (*n* = 8) from Greater Vancouver, British Columbia, Canada. *S.* Enteritidis FSL S5-483 was used as an indicator organism for phage isolation. Of the phages recovered, four broad-host range phages were isolated: SI1 (from irrigation water), SF1 (from cattle feces) and SS1 and SS4 (from sediment). Phage SI1 was isolated following direct plating by mixing 10 g of sample with 90 ml of salt-magnesium (SM) buffer (0.05 M Tris-HCl; 0.1 M NaCl and 0.01 M MgSO_4_; pH 7.5), followed by passage through a 0.45 μm membrane (Pall Corporation, Port Washington, NY, United States). Then, 100 μl of filtrate was mixed with 300 μl of 1:10 diluted *S.* Enteritidis grown to 16 h and four ml 0.7% TSA top agar, according to the double agar overlay method (Adams, 1959).

Phages SF1, SS1, and SS4 were isolated following enrichment by mixing 10 g of water, cattle feces or sediment samples, 90 ml TSB and 1 ml of *S.* Enteritidis cells grown to 16 h, followed by incubation at 37°C for 22 ± 2 h. The enriched samples were then spun at 4,000 × *g* and the supernatant subsequently passed through a 0.45 μm filter membrane (Pall Corporation). Then, 100 μl of filtrate was mixed with 300 μl of 1:10 diluted *S.* Enteritidis grown to 16 h and four ml 0.7% TSA top agar, according to the double agar overlay method (Adams, 1959).

Plates were incubated at 37°C for 18 ± 2 h for visualization of plaques. Plaques were lifted from the agar surface using a truncated pipette tip, suspended in 200 μl SM buffer, and rested for at least 6 h at room temperature. Double agar overlays were prepared with the suspension as described previously (Adams, 1959). Three single plaque isolations were carried out to obtain a pure phage lysate. Finally, phages were concentrated and stored at 4°C for further analyses.

### Phage Host Range Determination and Lysis from without

Prior to host range determination, phage lysates were standardized to a concentration of 10^9^ PFU/ml as recommended by Khan Mirzaei and Nilsson (2015). The host ranges of the phages were tested by spotting 5 μl of lysate, in duplicate, on a lawn of *Salmonella* host cells grown to 16 h (*n* = 61, **Table 1**). To test for the presence of lysis from without (LO), successive 10-fold dilutions of the phage were also prepared in sterile SM buffer and 5 μl of each dilution spotted in duplicate on the *Salmonella* lawn. Plates were incubated at 37°C for 18 ± 2 h. Zones of clearing were characterized with a scaling system as described by Kutter (2009), where 0 indicated a zone with complete turbidity (no lysis) and +4 indicated a completely clear zone with no turbidity (**Table 1**). LO was detected where complete lysis (+4 ranking) was observed at low dilutions (i.e., 10^-1^ PFU/ml), yet no lysis was observed at further dilutions (Kutter, 2009).

### Transmission Electron Microscopy

High titer phage lysates (10^9^ – 10^11^ PFU/ml) were chosen for transmission electron microscopy (TEM) and prepared for imaging as described previously (Deveau et al., 2006), with modifications. Briefly, one ml of lysate was spun at 4°C for 1.5 h at 21,000 × g. The supernatant was subsequently discarded and the last 100 μl was saved. Consequently, 1 ml of 0.1 M ammonium acetate (Amresco, Solon, OH, United States) was added and the suspension subsequently spun again at 4°C for 1.5 h at 21,000 × g. This purification was repeated twice, with the last 100 μl reserved for TEM.

For grid preparation, 3 μl of purified lysates were placed on carbon coated copper grids (Ted Pella, Redding, CA, United States) following glow-discharge. The phage preparations were subsequently negatively stained with 2% phosphotungstic acid (Ted Pella). A Hitachi H-7600 transmission electron microscope was used for acquiring the images at the University of British Columbia Bioimaging Facility. An accelerating voltage of 80 kV was used for imaging.

### Phage Genome Content Determination and Restriction Enzyme Analysis

Prior to nucleic acid extraction, DNase I (Invitrogen, Carlsbad, CA, United States) and RNAse A (Invitrogen) were added to high titer phage lysates (10^9^ – 10^11^ PFU/ml) to final concentrations of 10 and 55 μg/ml, respectively, for degradation of host nucleic acid (Merabishvili et al., 2014), followed by incubation at 37°C for 30 min. Phage nucleic acid was then extracted with the PureLink Viral RNA/DNA Mini Kit (Thermo Fisher) as per the manufacturer’s instructions. The concentration and quality of the extracted nucleic acid was determined with a Nanodrop spectrophotometer (Thermo Fisher), where an A260/280 ratio of ∼1.8 and A260/230 ratio of ∼2.0 were considered as pure.

Restriction enzyme analysis was conducted to confirm the unique identities of the phages. Nucleic acid was digested with *EcoR*1 (New England Biolabs, Ipswich, MA, United States) according to the manufacturer’s instructions. Subsequently, 10 μl volumes of the nucleic acid digests were loaded onto a 1% agarose gel (Amresco) and electrophoresed in 1X TAE buffer (Thermo Fisher) at 80 V for approximately 1 h. Band patterns were visualized using the ChemiDoc MP System (Bio-Rad Laboratories).

### Single Step Growth Curves

Single step growth curves were constructed to determine the phage burst sizes and burst times, according to Park et al. (2012), with modifications. Cultures of *S.* Enteritidis were grown for 16 h in TSB (37°C, 170 rpm). One ml of culture was then added to 9 ml of fresh TSB and incubated at 37°C at 170 rpm until an optical density at 600 nm (OD_600_) of 1.0 (∼10^9^ CFU/ml; stationary phase) was attained. Phages were then individually added at MOI of 0.01 and allowed to adsorb for 5 min at room temperature. To remove excess phage particles, the co-culture was spun at 4,000 × g at 4°C and the supernatant discarded. The pellets were resuspended in 10 ml of fresh TSB and incubated at room temperature with gentle agitation. Subsequently, 50 μl aliquots were collected every 5 min for a total duration of 60 min, immediately serially diluted in SM buffer, and spotted in duplicate on a host agar lawn of *S.* Enteritidis grown to 16 h for titer determination. Plates were incubated at 37°C for 18 ± 2 h for visualization of plaques. This experiment was independently conducted three times for each phage.

### Lysogeny Analysis

Resistant colonies of *S.* Enteritidis in the centers of spot assays (*n* = five colonies per phage) were selected to test for lysogeny. First, isolated colonies were serially re-streaked five times on TSA to reduce phage carry-over. On the fifth streak, a random colony was selected for polymerase chain reaction (PCR) to confirm their *Salmonella* identity by using primers specific to *invA*, according to Fong and Wang (2016). Briefly, a single colony was suspended in 200 μl of sterile de-ionized water and lysed in a microwave for two min. PCR detection was then carried out with the TopTaq Master Mix Kit (Qiagen, Valencia, CA, United States) with primers specific for the *invA* gene (forward: 5′-TCA TGG CAC CGT CAA AGG AAC C-3′ and reverse: 5′-GTG AAA TTA TCG CCA CGT TCG GGC AA-3′) (Li et al., 2012). PCR cycling conditions were as follows: initial denaturation (3 min, 94°C); three-step cycling, including denaturation (30 s, 94°C), annealing (30 s, 56°C), and extension (1 min, 72°C); followed by a final extension (10 min, 72°C). Sizes of the PCR products were confirmed with electrophoresis on 2% agarose (Amresco, Solon, OH, United States) with 1X Tris-acetate-EDTA (TAE) buffer (Thermo Fisher, Waltham, MA, United States). PCR products were visualized using the ChemiDoc MP System (Bio-Rad Laboratories, Hercules, CA, United States).

Colonies arising from the fifth streak were cultured to test for phage lysogeny as previously described (Petty et al., 2006). Colonies (*n* = 10) were suspended in 10 ml TSB and incubated at 37°C at 170 rpm for 20 h. Cultures were subsequently spun at 4,000 × g at 4°C to sediment the bacteria, and the supernatant tested for the spontaneous release of phage particles by spotting 5 μl in duplicate onto prepared *S.* Enteritidis agar overlays. Plates were incubated at 37°C for 18 ± 2 h for visualization of plaques. Supernatants from *S.* Enteritidis infection by Felix-O1, a strictly lytic phage, were used as a negative control.

### Temperature and pH Stability Assay

Phage lysates were diluted in TSB to an initial concentration of ∼10^7^ PFU/ml and subsequently stored at a range of temperatures (-20, 4, 22, and 37°C) for determination of relative temperature stabilities. Controls (no phage) were included for each temperature. The samples stored at -20°C were prepared in single-use 15 μl aliquots to prevent multiple thawing and freezing events throughout the assay. Temperature stability experiments were conducted three times for each phage.

To test pH stability of the phages, phage lysates were diluted to ∼10^8^ PFU/ml in TSB at varying pH ranges of pH 4.0, 6.0, 8.0, and 10.0 (adjusted with 6 M HCl or 6 M NaOH) and subsequently stored at room temperature for further analyses. Blank controls (no phage) were included for each pH. These pH and temperature ranges were chosen based on previously reported similar assessments (Thung et al., 2017) and reflect the various pH and temperature conditions encountered in produce production chains (Park et al., 2012; Rombouts et al., 2016). pH stability experiments were conducted three times for each phage.

Phage titers were assessed on days 2, 4, 8, 10, 14, 16, 20, 25, and 30. Briefly, 10 μl volumes were serially diluted in SM buffer and 5 μl spotted in duplicate on prepared top agar of *S.* Enteritidis grown to 16 h. Enumeration of plaques were determined after incubation at 37°C for 18 ± 2 h.

### Spectrophotometric Analysis of Phage Lysis Efficacy

Cultures of *S.* Enteritidis FSL S5-483, *S.* Agona FSL S5-513 and *S.* Typhimurium LMFS-JF-001 were prepared as described previously in section “Bacterial Strains and Growth Conditions.” Following incubation, cultures were spun at 4,000 × g and the cell pellets washed three times with fresh TSB. Then, the cultures were loaded into 96-well plates to a final concentration of 5 × 10^4^ CFU/ml and infected with phages SI1, SF1, SS1, and SS4 at MOIs of 1, 10, and 100 PFU/CFU. Plates were placed into a plate reader (SpectraMax M2, Molecular Devices, Sunnyvale, CA, United States) set to 25°C for determination of cell density at OD_600_ every 30 min for 36 h. Each experiment was independently conducted three times.

### Phage SI1 Biocontrol of *Salmonella* on Sprouting Alfalfa Seeds

The lysate of phage SI1 was tested for its efficacy to control *S.* Enteritidis on germinating alfalfa seed over 6 days. Six days was chosen as approximately 5–7 days are required for alfalfa seeds to sprout (Kramer and Lim, 2004). Cultures of *S.* Enteritidis grown to 16 h were spun at 4,000 × g for 10 min, washed three times with sterile potable water and serially diluted to a final volume of 35 ml of sterile water. The seed was inoculated by drop-wise addition of 15 ml diluted culture to 150 g seed to achieve an initial concentration of approximately log 3.5 CFU/g. Blank controls were processed similarly, but with sterile water only. The seed was placed in a biological safety cabinet at room temperature under continuous air flow for 2 h. Finally, the seed was transferred to sterile plastic boxes lined with a layer of sterile gauze pad and stored in the dark in a 22°C incubator. Three independent replicate experiments were performed.

The lysate of phage SI1 was applied to the seed at 22 h post-inoculation. Briefly, 75 g of inoculated seed was aseptically removed and treated with phage SI1 in 35 ml sterile water to yield an MOI of approximately 110 PFU/CFU. The seeds were soaked for 2 h at room temperature with gentle agitation by shaking at 175 rpm. Inoculated seed and controls that received only sterile water were processed in tandem. A 2 h phage soak was chosen due to (i) the short latent period (25 min) and relatively high burst size (83 phages) of SI1, thereby facilitating approximately four cycles of productive infection; and (ii) the simulation of a logistically feasible decontamination step, performed within a time frame that could be adopted into commercial sprout production practices. Following the soak, the excess fluid was removed by straining through sterile filter paper. Seed samples were then aseptically transferred to sterile plastic boxes lined with a layer of sterile gauze pad. Treated seed was stored in the dark in a 22°C incubator.

The germinating seed was moistened with seven ml of sterile water every 24 h over 6 days. Simultaneously, 10 g of seeds were removed daily and mixed with 100 ml of sterile phosphate buffered saline (PBS; Amresco) in a sterile Whirlpak bag (Nasco, Fort Atkinson, WI, United States). The samples were placed in a Stomacher (Seward, Worthing, West Sussex, United Kingdom) and homogenized for 2 min at 230 rpm. Subsequently, 100 μl aliquots were serially diluted in PBS and spread over xylose-lysine deoxycholate (XLD; Amresco) agar in duplicate. XLD plates were incubated at 37°C for 22 ± 2 h for enumeration of *Salmonella* (red colonies with black centers). Phage titers were measured by spotting 5 μl in duplicate on TSA seeded with *S.* Enteritidis grown to 16 h. Plates were incubated at 37°C for 18 ± 2 h for enumeration of plaques.

To assess the impact of the phage treatment on the final sprout yield, 150 g of seed was artificially contaminated with *S.* Enteritidis and 75 g was withdrawn for phage treatment, as per the procedures described previously. Sprouting seeds were then weighed after 6 days. Three independent replicate experiments were performed, with two technical replicates taken for each measurement.

### Statistical Analysis

For the pH and heat stability assays, the final titer of the phages after 30 days of treatment was compared to the initial titer at the beginning of treatment with a Student’s *t*-test (α = 0.05). To compare the relative susceptibilities of the phages to each treatment, the log decreases in phage titer after 30 days of treatment was calculated (i.e., the difference in log PFU/ml at time zero and after 30 days of treatment). A one-way analysis of variance (ANOVA) was then implemented with a Tukey’s Honest Significant Difference *post hoc* test applied to all significant ANOVA results (α = 0.05).

For the sprouts biocontrol assay, the log differences in *Salmonella* counts at each sampling time point between untreated and treated alfalfa seed samples were analyzed with a Student’s *t*-test with a significance level of α = 0.05. The differences in weights between the control and treated alfalfa sprouts were also assessed using a Student’s *t*-test (α = 0.05).

All statistical analyses were performed using JMP version 11.1.1 (SAS Institute, Inc., Cary, NC, United States). A *P*-value of ≤0.05 was considered statistically significant for all analyses.

## Results and Discussion

### Host Range Determination of *Salmonella* Phages and Detection of Lysis from without

Results of host range analysis of the four phages suggested that they were able to lyse 31–37% of the *Salmonella* strains tested as indicated by a +3 or +4 spot test (**Table 1**). Susceptible strains encompassed (i) the serotypes causing the highest rates of salmonellosis (i.e., *S.* Enteritidis and *S.* Typhimurium); (ii) serotypes involved in North American outbreaks (i.e., *S.* Agona, *S.* Saintpaul, and *S.* Heidelberg); and (iii) emerging, uncommon serotypes (i.e., *S.* Berta and *S.* Canada) (Ellis et al., 1998).

SI1, SS4, SF1, and SI1 were classified as broad host-range phages as they infected the largest proportion of the tested *Salmonella* strains, compared to other *Salmonella* phages recovered and characterized (*n* = 44). Of the 61 *Salmonella* strains tested, 23 were susceptible to infection by SI1, 22 were susceptible to SS4, 20 were susceptible to SF1 and 19 to SS1, as indicated by a +3 or +4 clearing (**Table 1**). The similarity between host ranges indicate that these four phages may recognize similar host receptors (Kalatzis et al., 2016). It should be noted that strains of *S.* Typhimurium (*n* = 8), responsible for the highest proportion of foodborne salmonellosis, demonstrated a high degree of susceptibility to these phages; seven were susceptible to infection by SI1 and SS4 and six were susceptible to SF1 and SS1. LO was not observed in any of the characterized phages, indicating the presence of obligate productive infection with all *S. enterica* strains tested.

### General Characterization

#### Phage TEM, Burst Size, Genotyping and Lysogeny

**FIGURE 1:**
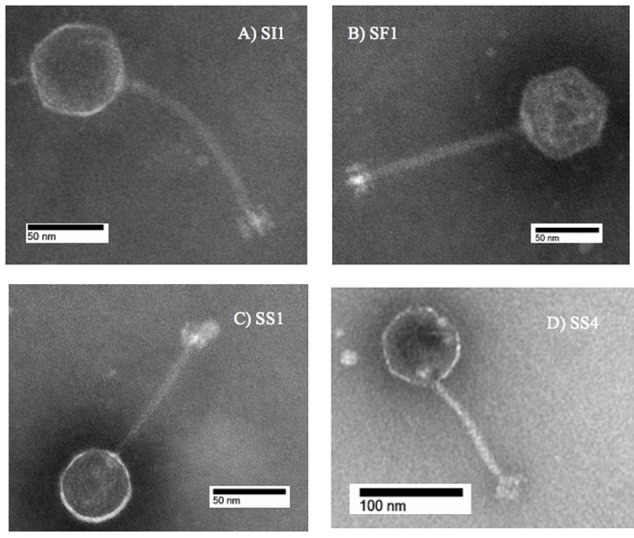
Transmission electron microscope images of **(A)** SI1, **(B)** SF1, **(C)** SS1, and **(D)** SS4.

SI1, SF1, SS1, and SS4 formed clear plaques on their host, *S.* Enteritidis, although they differed in size. SI1 and SS1 formed 1 mm clear plaques, but SS1 plaques also possessed slightly turbid haloes. Similarly, SS4 and SF1 formed larger plaques of 1.5 mm diameter, but SS4 plaques possessed slightly turbid haloes. It is suggested that halo formation is the result of endolysin secretion upon lysis of host cells (Cornelissen et al., 2012). Structural examination with TEM revealed their distinct morphologies, with all four phages belonging to the family *Siphoviridae* (**Figure 1**), consisting of rigid, non-contractile tails and double stranded DNA. SI1 is 207 ± 5 nm in length with a spherical head and a small appendage structure located at the crown. SF1 is 215 ± 3 nm long, possessing an icosahedral-shaped head. SS1 and SS4 exhibited structural similarity; both with spherical heads and 200 ± 2 nm and 202 ± 5 nm in length, respectively.

**FIGURE 2:**
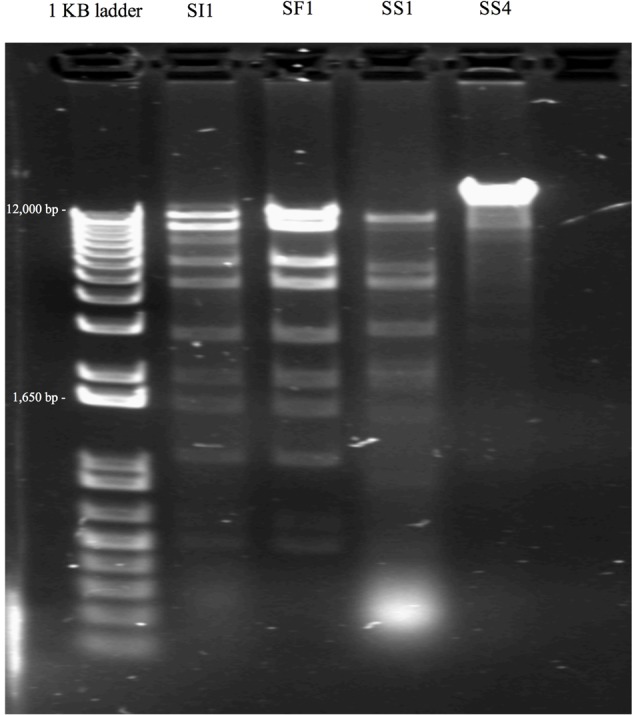
Restriction banding patterns of phage upon digestion by endonuclease *EcoR*I.

Subsequent typing of SI1, SF1, SS1, and SS4 was accomplished by restriction enzyme analysis. Digestion with *EcoR*1 yielded four distinct banding patterns, confirming the unique identities of these phages (**Figure 2**). It was found that SI1 has an approximate genome size of 87,000 bp, SF1 with a genome size of 80,800 bp, and SS1 and SS4 with substantially smaller genome sizes of 44,150 and 65,000 bp, respectively.

Some patterns only differed by a few bands, suggesting the conservation of *EcoR*1-specific cutting sites and a familial relationship between the characterized phages. Although Siphoviruses are known to exhibit remarkable mosaicism (Santander et al., 2017), it is expected that several components would exhibit notable similarity as these phages belong to the same family and present similar, though not identical, host ranges. Indeed, *Salmonella* phages fSE1C and fSE4C previously isolated from pickle sauce and ground beef, respectively, were digested with *EcoR*I, *Hind*III, and *Hae*III restriction enzymes and showed very similar banding patterns (Santander et al., 2017). Further analysis revealed a similarity of 43.09% between the genomes, with genes involved in structure, replication, host specificity and DNA metabolism showing remarkable conservation (Santander et al., 2017).

**FIGURE 3:**
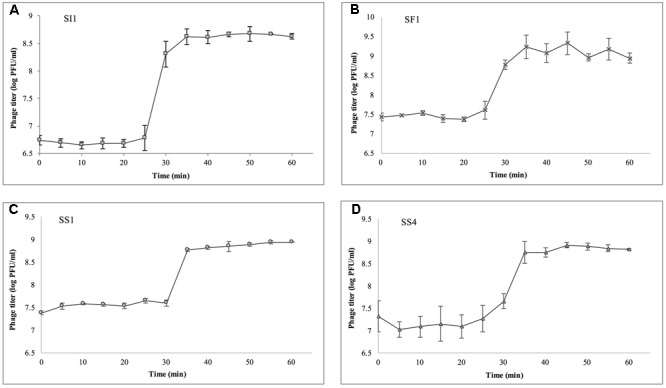
Single step growth curves of **(A)** SI1, **(B)** SF1, **(C)** SS1, and **(D)** SS4. Data shown are the mean of three replicates ± SD.

Single step growth curves were constructed to determine the infection potential of each phage (**Figure 3**). SI1, SF1, and SS1 possess latency periods of 25 min while SS4 possesses a latency of 30 min. The burst size of SI1 is 83 phages per infected cell, whereas the burst sizes of SF1, SS1, and SS4 are 45, 20, and 31 phages per infected cell. These phage infection parameters are in the range of those observed for Siphoviridae phages (Carey-Smith et al., 2006; Silva et al., 2014; Pereira et al., 2016b). For phage therapy in the food industry, it is often desirable to possess short latent periods and high burst sizes (Kalatzis et al., 2016), therefore the infection parameters outlined here demonstrate the potential of the characterized phages, particularly SI1, for use in biocontrol efforts.

Phages were tested for harborage of lysogenic elements by culturing phage-resistant colonies of *S.* Enteritidis and testing the supernatant for spontaneous release of phage particles. Absence of lysogenic integration into the host genome is a pre-requisite for phage biocontrol of food (Levin and Bull, 2004). No phage particles were detected upon spot-testing on overlays seeded with *S.* Enteritidis, indicating a strictly lytic life cycle and their suitability for use in phage therapy (Rombouts et al., 2016). Additionally, the production of clear plaques further confirmed their lytic life cycle.

#### pH and Temperature Stability

**FIGURE 4:**
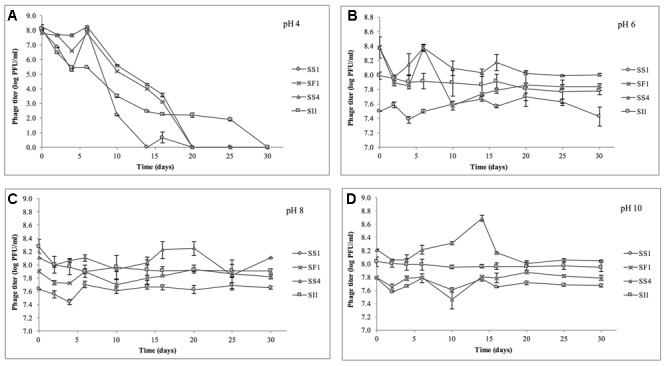
Stability of phages SI1, SF1, SS1, and SS4 at **(A)** pH 4, **(B)** pH 6, **(C)** pH 8, and **(D)** pH 10 over a period of 30 days. Data shown are the mean of three replicates ± SD.

At pH = 4, SF1, SS1, and SS4 were reduced to undetectable concentrations (<200 PFU/ml) by day 20 (**Figure 4**). SI1 was reduced to less than 200 PFU/ml by day 30. From day 6 onward, SS1 showed a more rapid decline than SF1 and SS4. These results suggest that of the four phages, SS1 would be the least stable biocontrol agent in acidic conditions, whereas SI1 would be the most stable.

In conditions ranging from pH = 6 – 10, no phage titer decreased by more than 0.544 ± 0.067 log PFU/ml, and only in one instance was titer (SS4 at pH 10) significantly (*p* < 0.05) reduced. On average, stability increased as the pH increased from pH 4 to pH 10, but there was some variability between phages. SI1 and SF1 were most stable at pH 10, but SS1 and SS4 were most stable at pH 8. SI1 was significantly (*p* < 0.05) less stable than each of SS1, SF1, and SS4 at pH = 8.

Our results are supported with previous findings of rapid declines in titer at pH 4.2 but only a gradual decline at pH 5.8 (Leverentz et al., 2001). However, Ahiwale et al. (2013) reported instability at pH = 10–12 of *Salmonella* phage. This inconsistency may be due to the structure of individual phages assayed. Having long flexible non-contractile tails, SF1, SS1, SS4, and SI1 belong to the family *Siphoviridae*, whereas phages assayed by Ahiwale et al. (2013) possessed short, stubby, non-contractile tails, representing *Podoviridae*. Similar to our phages, Hamdi et al. (2017) found that Siphovirus SH6 was unstable at pH 2–4 and stable at pH 5–11, whereas Myovirus SH7 was stable at pH 3–11. Differences in isoelectric points (pI) of the phages may also contribute to these differences in stability, particularly at acidic pH, as viral aggregation is common when pH ≤ pI and has previously led to decreases in titer of approximately three log PFU/ml (Langlet et al., 2007).

In the heat stability assay, nine of 16 phage titers were significantly (*p* < 0.05) reduced, but by no more than 1.0 ± 0.1 log PFU/ml over the range of temperatures tested, suggesting that SS1, SF1, SS4, and SI1 will retain the stability required for use as biocontrol agents at temperatures commonly encountered in produce production chains (Rombouts et al., 2016).

**FIGURE 5:**
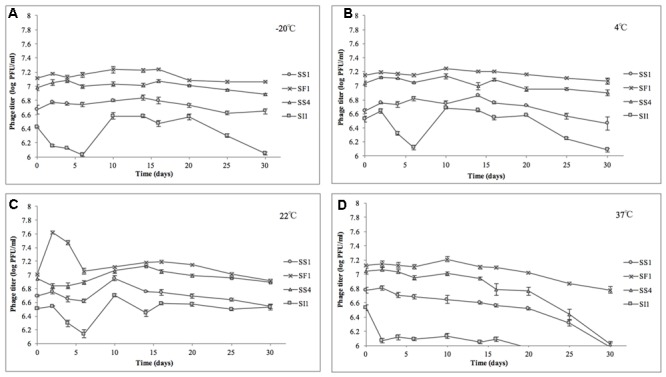
Stability of phages SI1, SF1, SS1, and SS4 at **(A)** –20°C, **(B)** 4°C, **(C)** 22°C, and **(D)** 37°C over a period of 30 days. Data shown are the mean of three replicates ± SD.

SS1, SF1, and SS4 were significantly (*p* < 0.05) more stable at -20, 4, and 22°C than at 37°C (**Figure 5**). From -20 to 22°C, there were no significant (*p* < 0.05) differences in stability between SS1, SF1, and SS4, but at 37°C, SF1 was significantly (*p* < 0.05) more stable than both SS1 and SS4. SI1 was detected to be most stable (*p* < 0.05) at a temperature of 22°C, and significantly (*p* < 0.05) more stable than SS1 at this temperature. All four phages were least stable at 37°C, and more stable at -20°C than 4°C. Aside from SI1 being most stable at 22°C, our results agree with previous reports in that Siphovirus stability decreases with an increase in temperature above 20°C (Jepson and March, 2004). Previous work has demonstrated the stability of Podoviruses to be most stable from 4 to 36°C, with highest stability retained at the lower end of the temperature spectrum (Ahiwale et al., 2013). This parallelism between phages of different families suggests that differences in phage tail morphologies may not be a main contributor to the variances in phage stability at different temperatures; however, Thorne and Holt (1974) have reported a negative correlation between temperature and tail contraction, and hence loss of activity, in *Myoviridae* phages. Given the importance of assaying for stability prior to adoption into the commercial market, these results indicate that their environmental stability makes these phages good candidates for use in biocontrol.

### Assessment of *in Vitro* Phage Infectivity in Tryptic Soy Broth

**FIGURE 6:**
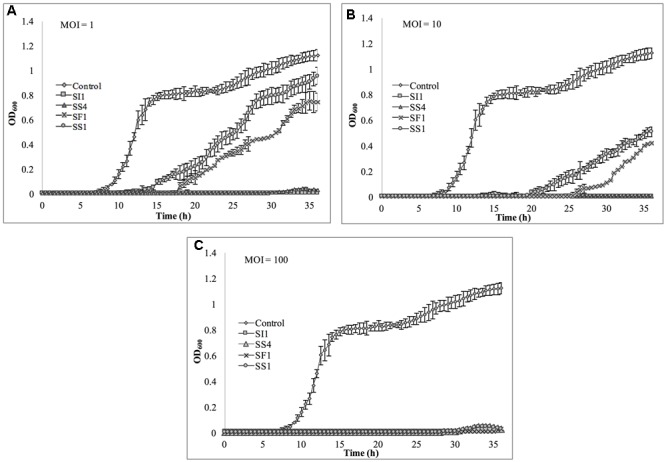
*In vitro* analysis of *S.* Enteritidis inhibition by phages SI1, SS4, SF1, and SS1 at **(A)** MOI = 1, **(B)** MOI = 10, and **(C)** MOI = 100. Data shown are the mean of three replicates ± SD.

The relative abilities of the four phages to suppress *S.* Enteritidis FSL S5-483, *S.* Agona FSL S5-513 and *S.* Typhimurium LMFS-JF-001 in TSB were assessed at an MOI of 1, 10, and 100 PFU/CFU at 25°C. At MOI = 1, both SI1 and SS4 suppressed the growth of *S.* Enteritidis over a 36 h period. Growth of *S.* Enteritidis was also suppressed by SF1 and SS1, but growth resumed at 13 and 17 h, respectively, after initial infection (**Figure 6**). Growth of *S.* Enteritidis appeared to recover at 31 h following treatment with SS4. An MOI of 10 prolonged the suppression of *S.* Enteritidis to 19 and 25 h when infected with SF1 and SS1, respectively. Further, application of SS4 at an MOI of 10 caused complete inhibition of *S.* Enteritidis growth for 36 h. Finally, phage treatment at an MOI of 100 suppressed the growth of *S.* Enteritidis for the 36 h duration (**Figure 6**).

**FIGURE 7:**
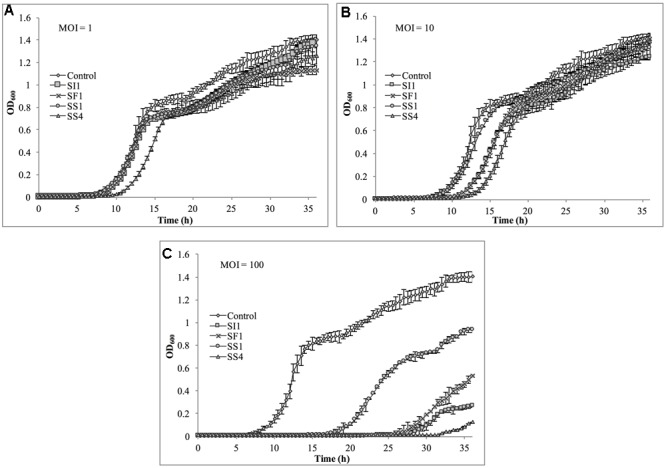
*In vitro* analysis of *S.* Agona inhibition by phages SI1, SS4, SF1, and SS1 at **(A)** MOI = 1, **(B)** MOI = 10, and **(C)** MOI = 100. Data shown are the mean of three replicates ± SD.

Suppression of *S.* Agona also occurred with all MOIs tested (**Figure 7**), but the extent was not as pronounced as with the host, *S.* Enteritidis. Instead, considerable suppression did not occur until an MOI of 100 was evaluated. At this MOI, it appeared that SS1 was the least effective in controlling *S.* Agona as growth resumed 17 h after the initial infection. In contrast, SI1 and SS4 were the most effective in suppressing growth, although *S.* Agona appeared to recover at 28 and 32 h after infection with these phages, respectively.

**FIGURE 8:**
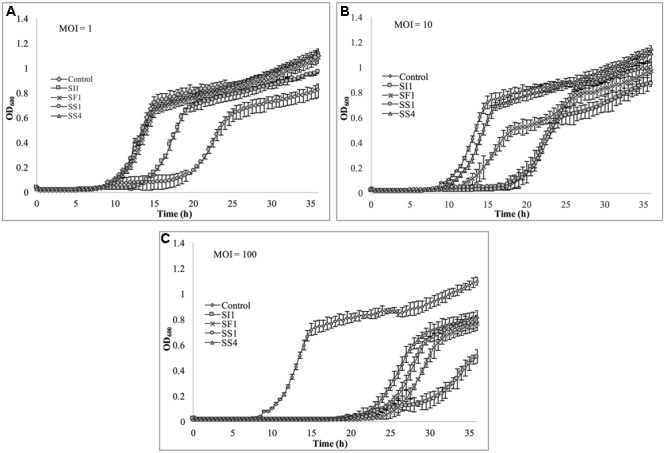
*In vitro* analysis of *S.* Typhimurium inhibition by phages SI1, SS4, SF1, and SS1 at **(A)** MOI = 1, **(B)** MOI = 10, and **(C)** MOI = 100. Data shown are the mean of three replicates ± SD.

Lastly, *S.* Typhimurium was tested for its susceptibility to the phages in TSB (**Figure 8**). Again, the extent of suppression after phage infection was not as pronounced as with *S.* Enteritidis, but did occur at all MOIs. The most dramatic reduction in growth occurred at MOI 100, although growth was not suppressed entirely for the 36 h duration. At this MOI, SI1 was the most effective in attenuating growth (**Figure 8**).

Although not all MOIs were effective in controlling growth of *Salmonella*, nor did all *Salmonella* strains show similar susceptibilities to the phages, it should be noted that phage infection at all MOIs, across all strains, resulted in an extended lag phase [defined as OD_600_ < 0.2 (Wang et al., 2009)], indicating that the phages had a suppressive effect on *Salmonella*. The ability of the *Salmonella* strains to recover after initial infection is likely due to the emergence of phage-resistant mutants (Guenther et al., 2012). It may be possible to prolong the duration of phage sensitivity by infection with a mixture of phages (Chan et al., 2013), though it was not evaluated in this present study.

With all strains, infection with an MOI of 100 proved to be the most effective and represents the MOI used for many food processing applications (Silva et al., 2014). However, with the phage host, *S.* Enteritidis, growth was completely suppressed at all MOIs with phage SI1, underlining its remarkable efficacy in controlling *S.* Enteritidis *in vitro*. Mechanistically, SI1 may require multiple attachment sites on the bacterial cell membrane for adsorption and/or SI1 receptor sites may be essential for cellular metabolic processes – both of which would contribute to the attenuation of phage resistance by the host (Rakhuba et al., 2010; Kong et al., 2011).

### Assessment of SI1 to Control *Salmonella* on Sprouting Alfalfa Seeds

The ability of SI1 to control *Salmonella* on sprouting alfalfa seeds was assessed. SI1, in particular, was selected for this study as it caused complete inhibition of *S.* Enteritidis in TSB at all tested MOIs and possessed the greatest burst size (approximately 83 phages) and possessed one of the shortest latent periods (25 min) (**Figure 2**). Moreover, *S.* Enteritidis is a serotype previously linked to North American sprout outbreaks (Centers for Disease Control and Prevention [CDC], 2017) and further, has been implicated in the highest number of salmonellosis outbreaks worldwide (Mattick et al., 2001). On the basis of these factors, they were selected for use in this biocontrol study.

**FIGURE 9:**
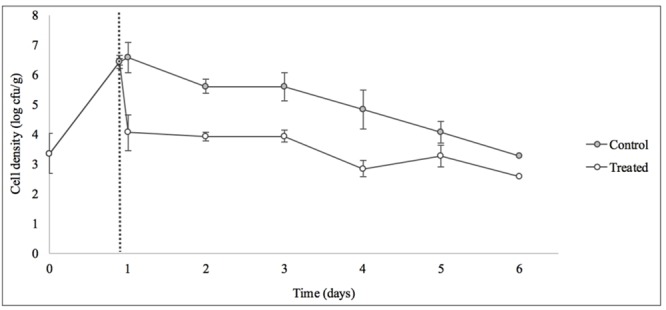
Control of *S.* Enteritidis on sprouting alfalfa seeds with phage SI1. Dotted line indicates the phage treatment at 22 h after artificial contamination of the seeds. Data shown are the mean of three replicates ± SD.

**FIGURE 10:**
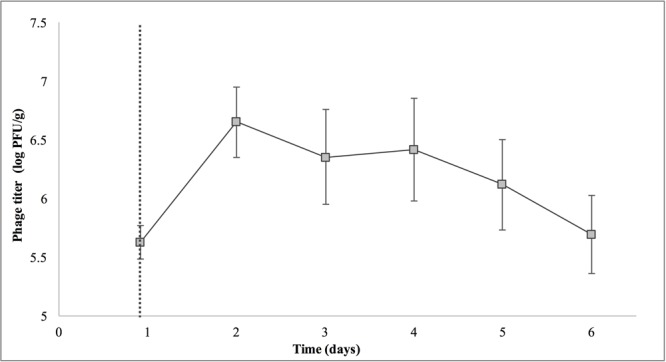
Titer of phage SI1 during control of *S.* Enteritidis on alfalfa sprouts. Dotted line indicates the phage treatment at 22 h after artificial contamination of the seeds. Data shown are the mean of three replicates ± SD.

Treatment with SI1 (MOI = 100) resulted in a significant (*p* < 0.05) 2.51 ± 0.24 log CFU/g reduction of *S.* Enteritidis, 2 h after treatment (**Figure 9**), corresponding to a decrease of 38.3 ± 3.0% of the initial viable population. This was accompanied by a 1.02 ± 0.33 log PFU/g increase in phage titer (**Figure 10**). In contrast, previous work by Kocharunchitt et al. (2009) reported a one log CFU/g decrease in *S.* Oranienburg populations following application of phage SSP6 onto alfalfa seeds at the beginning of germination. Similarly, a 1.37 log CFU/g reduction of *Salmonella* populations was observed on mustard seeds at 24 h following phage treatment (Pao et al., 2004). Sprout production standards, as set by Health Canada, recommend that sprout decontamination methods achieve a minimum three log reduction in pathogen counts (Canadian Food Inspection Agency [CFIA], 2014). Further validation of SI1 infectivity across a range of potential bacterial contaminants and at various stages throughout the sprouting process is therefore required to ensure complete compliance with Health Canada standards, although a >2.5 log CFU/g reduction of *S.* Enteritidis is promising. Additionally, the final weight of the seeds treated with *Salmonella* only (82.40 ± 2.83 CFU/g) was not significantly different (*p* > 0.05) than that of the phage-treated sprouts (80.64 ± 1.41 CFU/g), further demonstrating its potential suitability for use in industry.

On days 2–6 following phage treatment, *Salmonella* cell densities on treated alfalfa seeds were also reduced, but this was not significant (*p* > 0.05) (**Figure 9**). In line with this observation, phage titers increased the day of seed treatment, indicating its replication. However, the initial increase was followed by a stagnation of growth and small decreases in titer (**Figure 10**). Notably, it is presumed that the emergence of phage-resistant *Salmonella* may have contributed to the diminished effectiveness in the days following treatment. Indeed, phage-resistant *Salmonella* has been identified in both in *in vitro* systems (Vipra et al., 2013) and foods treated with phage (Kocharunchitt et al., 2009; Guenther et al., 2012). Emergence of bacterial mutants resistant to phage is particularly apparent when MOI values are high, as this enhances the selective pressure to resist infection (Vipra et al., 2013). It has been reported, however, that phage-resistant mutants possess attenuated pathogenicity and diminished fitness (Kong et al., 2011). A possible remedy to control the emergence of such mutants is through the use of a phage cocktail (Spricigo et al., 2013; Pereira et al., 2016a), which may additionally extend the spectrum of lysis to include other *Salmonella* strains (Chan et al., 2013).

Although the present results are not fully consistent with the data obtained *in vitro*, it is hypothesized that the simplicity of an *in vitro* system represents an ideal scenario for phage infection and multiplication. The nature of a food matrix presents with various complicating factors. For instance, possibilities include biofilm production on alfalfa sprouts (Kocharunchitt et al., 2009), which could hinder phage adsorption (Sutherland et al., 2004); growth of endogenous microbiota naturally present on sprout seeds, which may provide alternative adsorption sites (Ye et al., 2010); or internalization of *Salmonella* into the sprouts itself (Erickson, 2012), rendering them unavailable for phage attack. These factors could account for the diminished efficacy of the phage and also its inconsistent increases in phage titer throughout this assay. It is possible that additional phage treatments throughout the sprouting process, or phage treatment in combination with other treatments (e.g., chlorine or organic acid washes), would further reduce the viable *Salmonella* populations on alfalfa seeds. It should also be noted that the high initial load of *Salmonella* used in this assay is unrepresentative of real world situations, yet is important from a technical perspective to determine the log kill. Ye et al. (2010) reported a six log CFU/ml decrease of *Salmonella* on artificially contaminated mung bean sprouts upon treatment with a combination of six *Salmonella* phages and *Enterobacter asburiae*, a naturally competitive microorganism. Interestingly, this combination treatment was significantly more effective than treatment with phage or *E. asburiae* alone.

## Conclusion

Bacteriophage treatment of produce is an underdeveloped, emerging topic of interest and is currently not extensively used in industry. In this study, four lytic bacteriophages infecting *Salmonella* were assessed to determine their suitability for biocontrol in alfalfa sprout production. The results revealed that all four phages possessed desirable characteristics for use in biocontrol efforts. Among the phages characterized, SI1 proved to be particularly effective for control of *Salmonella* both *in vitro* and upon application onto sprouting alfalfa seeds. Although promising, future work should also aim to optimize this treatment, such as by incorporating hurdled treatments (i.e., with conventional sanitizers) or designing a multi-phage cocktail. Additionally, phage treatment of other sprouts varieties should be investigated to confirm the potential for use in related produce items.

## Author Contributions

PD, LG, RL, and SW were responsible for the study conception. KF and SW conceived the experimental design. AS designed the phage isolation protocol. KF and BL were responsible for data acquisition. KF isolated the bacteriophages in this study. LG and MD provided *Salmonella* strains used in this study. BL performed the pH and temperature stability analyses and participated in determination of the host range. KF carried out all other characterizations and performed the *in vitro* analysis and the biocontrol assay with alfalfa sprouts. KF, BL, and SW analyzed and interpreted the data. KF drafted the manuscript. All authors provided critical revisions and approved the manuscript.

## Conflict of Interest Statement

The authors declare that the research was conducted in the absence of any commercial or financial relationships that could be construed as a potential conflict of interest.
